# An Unsuspected Intraneural Perineurioma in a Pediatric Patient: A Case Report

**DOI:** 10.7759/cureus.103301

**Published:** 2026-02-09

**Authors:** Emmanuelle Tiongson, Benita Tamrazi, Debra Hawes

**Affiliations:** 1 Department of Pediatrics and Neurology, Children’s Hospital Los Angeles, Keck School of Medicine of the University of Southern California, Los Angeles, USA; 2 Department of Radiology, Children’s Hospital Los Angeles, Keck School of Medicine of the University of Southern California, Los Angeles, USA; 3 Department of Pathology, Children’s Hospital Los Angeles, Keck School of Medicine of the University of Southern California, Los Angeles, USA

**Keywords:** brachial plexus, nerve sheath neoplasm, pediatric neuropathy, perineurioma, peripheral neuropathy

## Abstract

Perineuriomas are rare tumors arising from perineurial cells that form the protective layer surrounding peripheral nerve fascicles. Four types of perineuriomas have been described: (i) intraneural, (ii) soft tissue (extraneural), (iii) sclerosing, and (iv) mucosal. Intraneural perineuriomas are rarely reported nerve sheath tumors that primarily affect the peripheral nerves of the upper and lower extremities.

In this report, we present a pediatric case in which the diagnosis of perineurioma was not suspected until lesional tissue was obtained, and the final pathologic diagnosis was made. The patient is a 17-year-old girl who presented with a three-year history of symptoms involving the left upper extremity, including weakness and cramping, which became progressively worse over time.

Diagnostic workup included magnetic resonance imaging (MRI), which showed enlargement and contrast enhancement of two of the left brachial plexus nerve trunks, suggestive of an inflammatory or infectious etiology, with schwannoma or neurofibroma also listed as less likely possibilities. An electromyogram (EMG) showed findings concerning for an anterior horn cell process, including amyotrophic lateral sclerosis (ALS). Nerve conduction studies (NCS) demonstrated axonal findings only in motor nerves, and needle EMG demonstrated denervation and fasciculations in multiple muscles. An initial biopsy of the brachial plexus was performed but was non-diagnostic. Ultimately, resection of the involved nerve trunks was performed. The diagnosis of intraneural perineurioma was not suspected preoperatively and was made only after histologic and immunohistochemical examination.

## Introduction

The perineurium is a smooth, transparent tubular membrane that surrounds nerve fascicles and comprises concentric layers of perineurial cells, separated by layers of collagen. Perineurial cells are the peripheral equivalent of the meningeal cells found intracranially. Perineuriomas are generally benign tumors that arise from the perineurial cells and are divided into four types: intraneural, soft tissue (extraneural), sclerosing, and mucosal [1].

Intraneural and soft tissue types are rare and typically account for an estimated 1% of all nerve sheath and soft tissue neoplasms [2]. Intraneural perineuriomas are far rarer than soft tissue perineuriomas, with approximately 172 cases reported [3]. The intraneural type has been seen more commonly in adolescents, without a sex predilection, and demonstrates a slow-growing, indolent behavior that can cause a mononeuropathy. This can affect large spinal nerve roots, trunks, or branches, including the brachial plexus, and has been rarely shown to occur in cranial nerves [2]. In one review of the literature [3], perineuriomas were found in the brachial plexus in 12.2% of cases; the same percentage (12.2%) was found in the radial nerve. More common locations were the sciatic nerve or its branches (41.2%) and the medial nerve (13.5%). These lesions can grow to more than 10 cm and are known to cause a fusiform or segmental enlargement of the nerve. They were first described in 1964 by Imaninariojda et al. [4] as a lesion in the right radial nerve of an 18-year-old patient. At that time, this lesion was thought to be a reactive process and was diagnosed as a hypertrophic neuritis.

Indeed, for many years, perineuriomas were thought by many to be reactive processes and subsequently were known by various names, including “localized hypertrophic neuropathy,” “intraneural neurofibroma,” “hypertrophic interstitial neuritis,” and “pseudo-onion bulb neuropathy” [3,5,6]. Clinically, these lesions usually present as a pure motor neuropathy or, less commonly, as a motor neuropathy with some sensory deficits [6]. A study published by Emory et al. [7], in which they looked at eight cases of perineurioma, concluded that perineuriomas represented a clonal neoplastic lesion, in which they found one case with abnormalities in chromosome 22, and that while most tumors remained localized, some showed extensive intraneural growth. Another study has shown chromosome 22 abnormalities in both intraneural and extraneural perineuriomas [8]. Soft tissue perineuriomas are not usually associated with nerves and histologically demonstrate a storiform, whorled appearance, with slender spindled cells and wavy nuclei. They are usually solitary and well circumscribed, without encapsulation, and can be collagenous, with a myxoid component. Soft tissue perineuriomas can also have a higher mitotic count than the intraneural type, with degenerative nuclear features and potential infiltration into the surrounding soft tissue, but these features have not been shown to be clinically significant [1,9].

The sclerosing type is found most commonly in the hands and fingers of young adults. Mucosal perineuriomas are commonly found in the colorectal region of the GI tract and grossly resemble a polyp [1].

## Case presentation

The patient was a 17-year-old right-hand-dominant female with a history of progressive wasting and weakness of the left hand and wrist that had begun nearly three years prior, when she was 15 years old. Her age at presentation was on the younger side, with the median age of onset of symptoms being 31.2 years and the range being 15-64 years. At that time, she had noticed difficulty fastening her seatbelt, which resulted in cramping of the left hand. Physical examination showed reduced muscle bulk at the left triceps brachii muscle and in the extensor and flexor compartments (extensor > flexor) of the left arm, compared to the right. Also noted was significant atrophy of the interossei muscles, with flattening of the thenar and hypothenar eminences. The tone of the left arm was also decreased compared to the right arm, with reduced muscle strength involving her left upper extremity (Table 1). She reported feeling fasciculations in the neck, face, left arm, and right leg on occasion, but this was not directly observed during the physical examination.

**Table 1:** Upper extremity strength according to the Medical Research Council (MRC) grading scale Source: Medical Research Council (MRC) grading scale [10] WF, Wrist Flexion; WE, Wrist Extension; FF, Finger Flexion; FE, Finger Extension; IO, Interossei; ThAb, Thumb Abduction

	Deltoid	Bicep	Tricep	WF	WE	FF	FE	IO	ThAb
R	5	5	5	5	5	5	5	5	5
L	5	5	3	4	2	4	2	2	2

Over time, she developed wrist drop. Sensation was preserved along the radial, median, and ulnar nerves. She also experienced increasing hoarseness of her voice, which started at approximately the same time as her left upper extremity symptoms. These clinical findings, except for the hoarseness, are consistent with those typically encountered in brachial plexus perineuriomas, which classically present as unilateral weakness and atrophy in the shoulder, arm, or hand. Our patient did not experience sensory deficits; however, sensory changes, including numbness, tingling, or sometimes even pain, were reported. The hoarseness initially raised the possibility of bulbar weakness associated with amyotrophic lateral sclerosis (ALS), which was in the differential diagnosis. Ultimately, it was not attributed to the perineurioma, but rather was thought to be surgically related (e.g., extubation/intubation and surgical approach). The patient's workup included electromyography (EMG) and nerve conduction velocity (NCV) testing. The EMG showed electrodiagnostic evidence of active and chronic neurogenic findings in the left extensor digitorum brevis (EDB) muscle, indicating a more active, subacute course than a chronic course of the patient's wrist drop, with only chronic findings in the abductor pollicis brevis (APB) muscle, which had undergone considerable atrophy. There was no evidence of widespread fasciculation or motor-predominant neuropathy in the remainder of the examination, which was normal in the right upper and lower extremities, as well as in the tongue. These findings were not suggestive of more widespread motor neuron disease, though she had prominent fasciculations present on her previous left upper extremity study. The differential diagnosis included denervation of the left upper extremity due to a focal brachial plexus lesion versus a multifocal anterior horn cell process, including Hirayama disease, due to the presence of fasciculations on the previous needle EMG examination. In retrospect, this may have represented her profound denervation changes. The more recent EMG findings are summarized in Table 2.

**Table 2:** Electromyogram (EMG) results The grading scale for the EMG results is 0 to 4+, indicating the frequency or prevalence of the abnormal electrical activity observed with the needle electrode: 0: No spontaneous activity observed. 1+: Fibrillations or positive sharp waves are observed in at least two sites within the muscle. This is considered minimal severity. 2+: Activity is observed with about half of the movements of the needle electrode. This indicates a moderate degree of abnormality. 3+: Activity is observed following almost every movement of the needle. 4+: Profuse activity where the baseline electrical signal is not visible due to the continuous presence of abnormal waves. MUAP, Motor Unit Action Potential; IA, Insertional Activity; Fib, Fibrillations; PSW, Positive Sharp Waves; Fasc, Fasciculations; AMP, Amplitude; Dur, Duration; PPP, Polyphasic Potentials; Poor Act, Poor Activation; CRD, Complex Repetitive Discharges

Muscle	Nerve	Roots	Spontaneous				MAUP			Recruitment
			IA	Fib	PSW	Fasc	Amp	Dur	PPP	Pattern
R. Deltoid	Axillary	C5-C6	N	None	None	None	N	N	N	N
R. Biceps brachii	Musculocutaneous	C5-C6	N	None	None	None	N	N	N	N
R. Triceps Brachii	Radial	C6-C8	N	None	None	None	N	N	N	N
R. Pronator teres	Median	C6-C7	N	None	None	None	N	N	N	N
R. First dorsal interosseous	Ulnar	C8-T1	N	None	None	None	N	N	N	N
R. Vastus lateralis	Femoral	L2-L4	N	None	None	None	N	N	N	N
R. Tibialis anterior	Deep peroneal	L4-L5	N	None	None	None	N	N	N	N
R. Gastrocnemius	Tibial	S1-S2	N	None	None	None	N	N	N	Poor Act
L. Extensor digitorum	Radial	C7-C8	CRD	1+	1+	None	1+	2+	N	Reduced
L. Abductor pollicis brevis	Median	C8-T1	N	None	None	1+	2+	2+	N	Reduced
L. Genioglossus	Hypoglossal	Medulla	N	None	None	None	N	N	N	N

Magnetic resonance imaging (MRI) demonstrated fusiform enlargement and contrast enhancement of the middle and inferior trunks of the left brachial plexus, suggestive of an inflammatory or infectious etiology. Neurofibroma or schwannoma was also considered a less likely possibility. 

An open biopsy was performed, but revealed only minimal chronic inflammation on pathologic examination. As the diagnosis remained undetermined, the patient underwent a further battery of studies. Genetic testing proved negative for ALS, neuromuscular disorders, and comprehensive neuropathies. MRI of the brain and spine was similarly negative.

**Figure 1:**
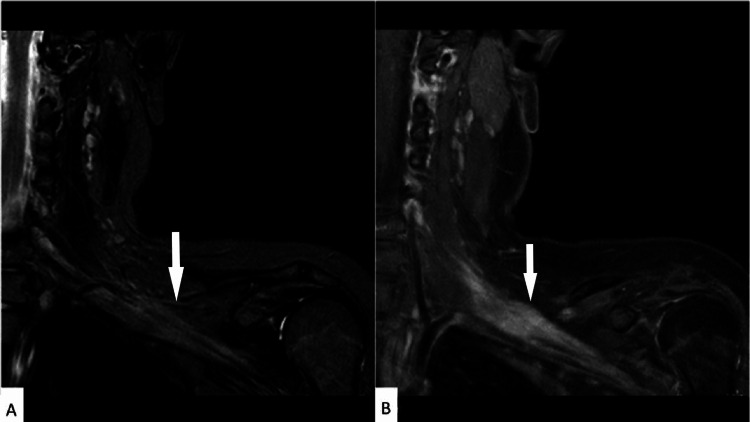
Magnetic resonance imaging of brachial plexus A) Coronal short-T1 inversion recovery (STIR) sequence demonstrates fusiform enlargement of the left middle and inferior trunks of the brachial plexus. The arrow points to an ill-defined thickening of the brachial plexus. B) Coronal T1 post-contrast with fat saturation sequence demonstrates corresponding enhancement. The arrow points to an ill-defined enhancement of the brachial plexus.

The patient continued to experience progressive wasting and weakness of the left hand and wrist. A repeat MRI was performed, which was unchanged from the initial MRI and showed a persistent fusiform mass along the middle and inferior trunks, with avid contrast enhancement (Figure 1). A second surgery was undertaken to re-explore the brachial plexus, with excision of lesional tissue and immediate reconstruction. At the time of surgery, it was confirmed that the nerve in question had no appreciable function, and the decision was made to excise the involved areas of the brachial plexus and reconstruct them with interpositional nerve grafts. The excised nerve was over 10 cm in length and consisted of the middle and lower trunks of the brachial plexus, all of which showed an enlarged diameter, making it larger than many perineuriomas. A study reported lengths of lesions varying from 0.3 to 20.0 cm, with most cases ranging from 1.5 to 7.0 cm [8]. The specimen was serially sectioned and entirely submitted for histologic evaluation. 

**Figure 2:**
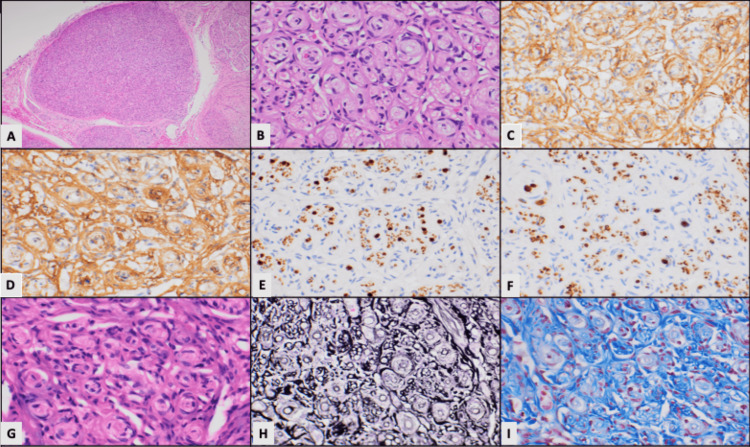
Pathologic findings A) H&E stain at 100x magnification reveals multiple enlarged fascicles with increased cellularity and variable fibrosis, with one fascicle prominent on cross-section in this image. B) H&E stain at 400x magnification reveals neoplastic perineurial cells forming concentric layers around axons. C) EMA IHC staining reveals positive membranous staining of perineurial cells. D) GLUT1 IHC staining reveals positive staining of neoplastic perineurial cells. E) S100 IHC stain highlights the Schwann cell component of the axon. F) Neurofilament protein IHC stain highlights the axons. G) Lack of staining with Luxol fast blue demonstrates a lack of myelin. H) Jones silver stain outlines the fibrosis in concentric rings (black) around the neoplastic cells. I) Masson trichrome stain reveals the extensive fibrosis (blue). H&E, Hematoxylin and Eosin; EMA, Epithelial Membrane Antigen; GLUT1, Glucose Transporter-1; IHC, Immunohistochemistry

On microscopic examination, virtually the entire length of the nerve trunks was found to be involved by the tumor, which consisted of enlarged fascicles with increased cellularity and variable fibrosis. On high magnification, it was evident that the enlarged fascicles were diffusely packed by a proliferation of neoplastic perineurial cells, forming concentric layers around axons (pseudo-onion bulbs). The perineurial cells were elongated and flattened, with minimal nuclear atypia and a delicate chromatin pattern. There were no areas of increased mitotic activity or necrosis. Immunohistochemically, the neoplastic cells were positive for epithelial membrane antigen (EMA) and glucose transporter-1 (GLUT1), while S100 highlighted the Schwann cells (Figures 2A-2I). The histologic features were diagnostic of an intraneural perineurioma. Tumor tissue was sent for molecular analysis, which revealed no significant gene fusions, and no variants or gene amplifications were detected. In aggregate, the combination of a three-year, purely motor mononeuropathy, fusiform T2-hyperintense enlargement of the lower brachial plexus, and EMA- and GLUT1-positive pseudo-onion bulbs fulfills diagnostic criteria for intraneural perineurioma.

The patient was seen in follow-up in our multidisciplinary brachial plexus clinic, with continued pain and progressive weakness. Gabapentin, in escalating doses, was not effective for her neuropathic pain symptoms. She continued to be quite limited in her activities of daily living (ADLs), given the involvement of her dominant hand. She has elected not to continue with medication titrations and not to have further surgical follow-up at our institution. She has benefited from occupational therapy sessions only in adaptations, but not necessarily in the return of muscle bulk or strength.

In light of the outcomes of this case, it is important to stress that the diagnosis of an intraneural perineurioma is benign and potentially non-surgical. The wide excision of this young woman’s brachial plexus resulted in more disability than the tumor alone.

## Discussion

Intraneural perineuriomas are rare nerve sheath neoplasms, with approximately 172 cases reported in the literature, and thus are often not immediately considered in patients presenting with symptoms of neuropathy. In addition, tissue from either a biopsy or resection is required for definitive diagnosis, which has led many to wonder if these lesions are underdiagnosed and, hence, underreported. Here, we present a case of an adolescent female with a history of neuropathy, in which the diagnosis of intraneural perineurioma was not considered preoperatively and was made only after resection of the lesion and subsequent pathologic examination.

By MRI, intraneural perineuriomas have fairly consistent findings, including being hypointense to isointense on T1-weighted MRI images and hyperintense on T2-weighted MRI images, with fusiform, segmental enlargement of the affected nerve [3,11]. Histologically, this enlargement generally corresponds to proliferation of predominantly or exclusively concentric whorls of spindle-shaped perineurial cells, the so-called pseudo-onion bulbs, with or without interspersed collagen fibers [2,6]. In addition, ultrasonography and ultrasound elastography have increasingly been applied in the assessment of peripheral nerves. These noninvasive imaging techniques allow evaluation of nerve morphology and stiffness, and may aid in the early detection and characterization of lesions such as perineuriomas [12,13]. 

True onion bulbs are defined as whorls of Schwann cell processes. Since the layered spindle cells in pseudo-onion bulbs are perineurial in origin, they can be differentiated from Schwann cells by positive immunohistochemical staining for EMA and GLUT1, in a membranous pattern, and only the central axon-Schwann cell complex will stain for S100 [2,6,14,15]. Schwann cell proliferations will immunoreact more diffusely for S100 protein. 

These tumors are benign, and the intraneural type has not been known to transform or metastasize. Mitotic figures may be rarely present, and the Ki-67 labeling index is not usually elevated. Mild elevation of mitotic activity, if present, does not appear to have any clinical significance [2,3,6,11]. Myelination and axon density vary from early to late lesions, as more perineurial layers and collagen fibrosis accumulate in the lesion, eventually decreasing the amount of normal myelin around the axons. Luxol fast blue will show the decreased myelination in the lesion, as it enters a later phase. Trichrome stain is helpful to highlight the dense collagen deposition in the later phases of the lesion. Intraneural perineuriomas can show abnormalities in the long arm of chromosome 22, but a definitive association with neurofibromatosis type 2 (NF2) has not been established [2,3]. This molecular finding is helpful to support a neoplastic process, but is not necessary. Denervation is seen on EMG and correlates with muscle atrophy. The diagnosis is confirmed by biopsy.

The differential diagnosis includes true onion bulb entities that are composed of Schwann cells. The following disease processes typically have their own unique clinical context, but can demonstrate the true onion bulb formations, which can enter the intraneural perineurioma differential diagnosis, including chronic inflammatory demyelinating polyneuropathy (CIDP), Charcot-Marie-Tooth (CMT) disease, and infantile demyelinating or hypomyelinating neuropathies, also known as Dejerine-Sottas disease. Conditions such as CMT have true onion bulbs seen histologically in cross-sections of the nerve, which form as a result of repeated episodes of demyelination and remyelination of the axon, leading to Schwann cells forming rings around individual axons [6,16]. These onion bulbs look similar to those seen in CIDP, which has a multifocal distribution in the body and is associated with many inflammatory diseases, like Guillain-Barré syndrome. CIDP also has phases of recurring demyelination and remyelination, with inflammation that can reveal large hypertrophic onion bulbs made of Schwann cells. These true onion bulbs will stain positively for S100 and will be negative for EMA and GLUT1, the reverse pattern of that seen in a perineurioma. It is rare to have a reactive intraneural perineurial proliferation, but it can be seen in the tongue and is characterized by hyalinized pseudo-onion bulbs. Genetic testing on this patient confirmed no common mutations associated with CMT disease or other genetic neuropathies.

## Conclusions

This patient was operated on and underwent a resection of the tumor before the definitive diagnosis was known. This surgery resulted in a noticeable decrease in function, as compared to presurgery. In retrospect, non-surgical management (e.g., MRI surveillance) or a smaller excisional open biopsy (as opposed to a more extensive resection) may have resulted in improved clinical outcomes. Increased awareness of intraneural perineurioma in the adolescent female population may change management and outcomes for future patients, by nature of being considered in the differential of new-onset brachial plexopathy in a patient without previous tumor history or a neurofibromatosis diagnosis.
